# Supplementary material for: Alpha 1 Antitrypsin is an Inhibitor of the SARS-CoV-2–Priming Protease TMPRSS2
Source: Pathog Immun. 2021 Apr 26;6(1):55–74. doi: 10.20411/pai.v6i1.408 (PMC8097828; doi:10.20411/pai.v6i1.408)
Supplement: Supplementary Figure 1 [file pai-6-055-s01.pdf]

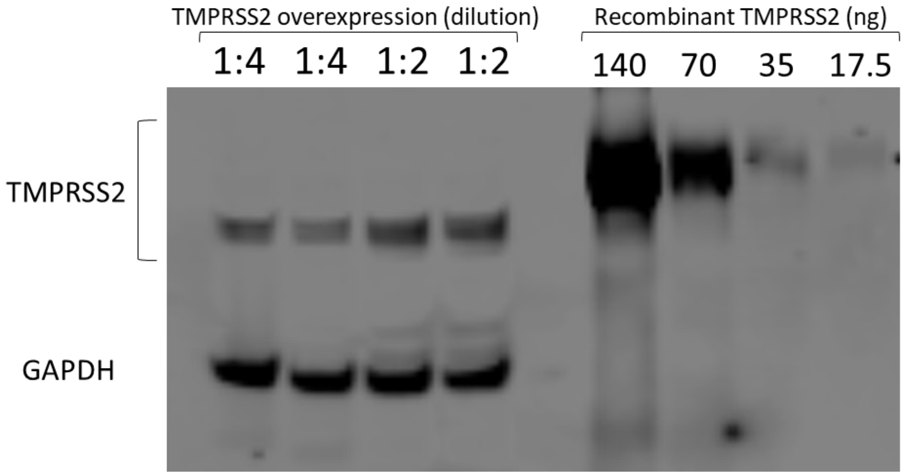

Supplementary Figure 1. TMPRSS2 protein quantity calculation. Western blot analysis of protein lysates from TMPRSS2-overexpressing cells (transfected HEK-293T) that were diluted 1:4 or 1:2 and of recombinant TMPRSS2 as indicated. GAPDH, glyceraldehyde-3-phosphate dehydrogenase; TMPRSS2, transmembrane serine protease 2.
