# Supplementary material for: Alpha 1 Antitrypsin is an Inhibitor of the SARS-CoV-2–Priming Protease TMPRSS2
Source: Pathog Immun. 2021 Apr 26;6(1):55–74. doi: 10.20411/pai.v6i1.408 (PMC8097828; doi:10.20411/pai.v6i1.408)
Supplement: Supplementary Figure 2 [file pai-6-055-s02.pdf]

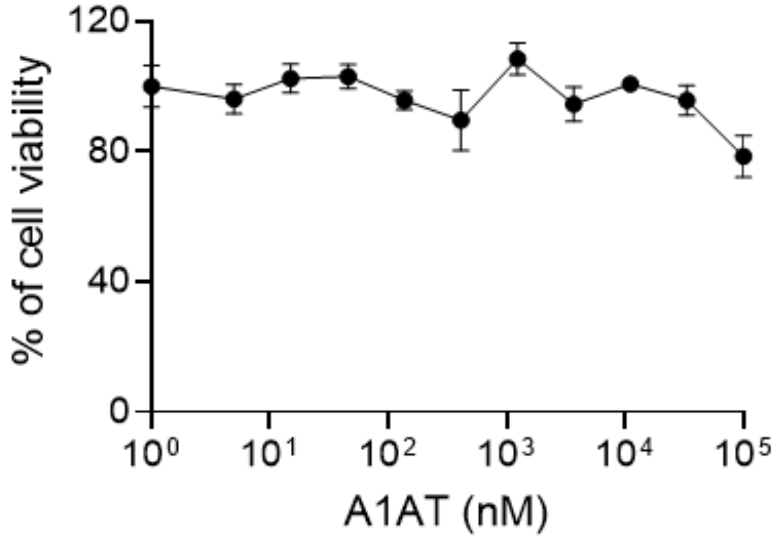


**Supplementary Figure 2. Viability assay.** HEK-293T cells were assessed according to their viability after 18 hours at the indicated concentrations of A1AT. Cell viability was calculated as the percent of viability compared to untreated cells. Results are presented as the mean ± SE of 2 independent experiments performed in 6 replicates. A1AT, alpha 1 antitrypsin.
