# Supplementary material for: Alpha 1 Antitrypsin is an Inhibitor of the SARS-CoV-2–Priming Protease TMPRSS2
Source: Pathog Immun. 2021 Apr 26;6(1):55–74. doi: 10.20411/pai.v6i1.408 (PMC8097828; doi:10.20411/pai.v6i1.408)
Supplement: Supplementary Figure 3 [file pai-6-055-s03.pdf]

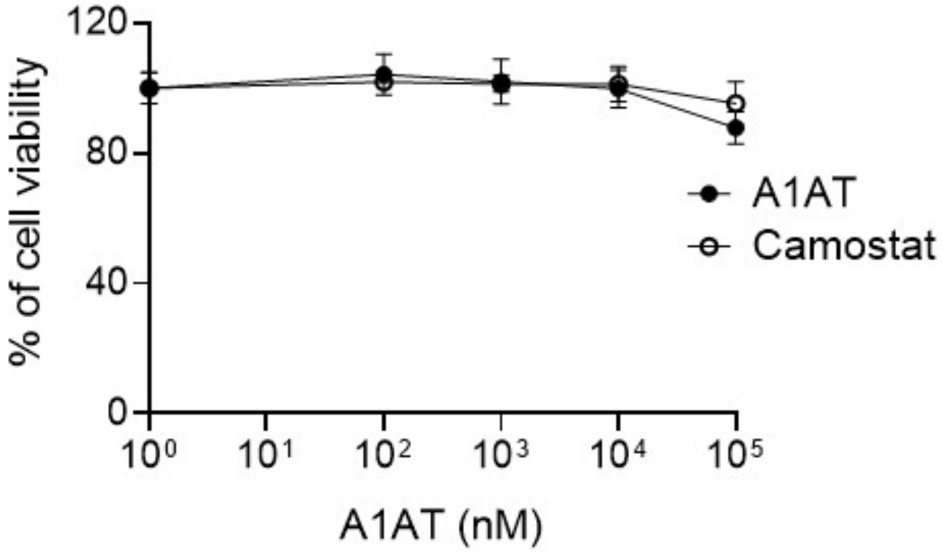

Supplementary Figure 3. Viability assay of A1AT and camostat on SARS-CoV-2 genomic copies. Caco-2 cells were assessed according to their viability at the indicated concentrations of A1AT. Cell viability was calculated as the percentage of viability compared to untreated cells. Results are the mean ± SE. A1AT, alpha 1 antitrypsin; camostat, camostat methylate.
